# Regular consumption of cod liver oil is associated with reduced basal and exercise-induced C-reactive protein levels; a prospective observational trial: A NEEDED (The North Sea Race Endurance Exercise Study) 2014 sub-study

**DOI:** 10.1186/s12970-021-00437-1

**Published:** 2021-06-28

**Authors:** Mette Wærstad Hansen, Stein Ørn, Christine B. Erevik, Magnus Friestad Bjørkavoll-Bergseth, Øyvind Skadberg, Tor H. Melberg, Kristin M. Aakre, Øyunn Kleiven

**Affiliations:** 1 Cardiology Department, Stavanger University Hospital, PO 8400, 4068 Stavanger, Norway; 2 Department of Electrical Engineering and Computer Science, University of Stavanger, Stavanger, Norway; 3 Department of Biochemistry, Stavanger University Hospital, Stavanger, Norway; 4 Department of Clinical Science, University of Bergen, Bergen, Norway; 5 Department of Medical Biochemistry and Pharmacology, Haukeland University Hospital, Bergen, Norway; 6 Department of Heart Disease, Haukeland University Hospital, Bergen, Norway

**Keywords:** Omega-3, Cod liver oil, Supplement use, Athletes, Exercise-induced inflammation

## Abstract

**Background:**

Dietary supplement use among recreational athletes is common, with the intention of reducing inflammation and improving recovery. We aimed to describe the relationship between omega-3 fatty acid supplement use and inflammation induced by strenuous exercise.

**Methods:**

C-reactive protein (CRP) concentrations were measured in 1002 healthy recreational athletes before and 24 h after a 91-km bicycle race. The use of omega-3 fatty acid supplements was reported in 856 out of 1002 recreational athletes, and the association between supplement use and the exercise-induced CRP response was assessed.

**Results:**

Two hundred seventy-four subjects reported regular use of omega-3 fatty acid supplements. One hundred seventy-three of these used cod liver oil (CLO). Regular users of omega-3 fatty acid supplements had significantly lower basal and exercise-induced CRP levels as compared to non-users (*n* = 348, *p* < 0.001). Compared to non-users, regular users had a 27% (95% confidence interval (CI): 14–40) reduction in Ln CRP response (unadjusted model, *p* < 0.001) and 16% (95% CI: 5–28, *p* = 0.006) reduction after adjusting for age, sex, race duration, body mass index, delta creatine kinase, MET hours per week, resting heart rate and higher education. CLO was the primary driver of this response with a 34% (95% CI: 19–49) reduction (unadjusted model, *p* < 0.001) compared to non-users. Corresponding numbers in the adjusted model were 24% (95% CI: 11–38, *p* < 0.001).

**Conclusion:**

Basal CRP levels were reduced, and the exercise-induced CRP response was attenuated in healthy recreational cyclists who used omega-3 fatty acid supplements regularly. This effect was only present in regular users of CLO.

**Trial registration:**

NCT02166216, registered June 18, 2014 – Retrospectively registered.

**Supplementary Information:**

The online version contains supplementary material available at 10.1186/s12970-021-00437-1.

## Background

Prolonged strenuous physical exercise induces inflammation [1, 2]. An increased exercise-induced inflammation is associated with attenuated recovery and exercise-induced muscular damage and soreness [3]. The use of omega-3 fatty acid supplements has been associated with a reduction in inflammatory markers in both recreational and elite athletes in several small-scale randomized trials [4]. However, some studies have reported no effect of omega-3 fatty acid supplements on inflammatory markers following exercise [5, 6]. These diverging findings may relate to type, intensity and duration of exercise, the population studied, timing of sampling and the type of inflammatory markers used to assess the response [7].

Omega-3 fatty acid supplements attenuate inflammation by modulating cell membrane function, down-regulating pro-inflammatory cytokines (such as IL-6), reducing production of arachidonic acid derivatives and reactive oxygen species [8]. Omega-3 fatty acid supplements contain a variety of eicosapentaenoic acids (EPA, 20:5n-3) and docosahexaenoic acids (DHA, 22:6n-3), and these substances have different effects on the inflammatory response. EPA is a precursor of leukotriens, attenuating cyclooxygenase 2 and arachidonic metabolism, while DHA is the precursor for protectins [8]. These differences might be of importance when evaluating the effect of omega-3 fatty acid supplements. In Norway, a supplement made from fresh arctic cod liver oil (CLO, “Tran”) has traditionally been a popular omega-3 fatty acid supplement. There is limited data on the relationship between the use of omega-3 fatty acid supplements and inflammation in recreational athletes, and particularly on the use of CLO.

The primary aim of this study was to determine the relationship between regular consumption of omega-3 fatty acid supplements and the exercise-induced inflammatory response in a large population of healthy recreational athletes. The secondary aim was to determine if there is a difference in the inflammatory response comparing CLO (“Tran”) and other types of omega-3 fatty acid supplements. C-reactive protein (CRP) was used to assess the inflammatory response, as CRP is a robust down-stream inflammatory marker of cytokines such as IL-6 [9]. All data was acquired during the same event, from a limited geographical territory to ensure as homogeneous population as possible.

## Methods

The North Sea Race Endurance Exercise Study (NEEDED) is a prospective, observational study that sampled data from 1002 presumably healthy recreational athletes in conjunction with a 91 km mountain bike race (The North Sea Race) in 2014. The study population consisted of individuals who were physically active but who did not train for competition at the same level of intensity and focus as competitive athletes. As the focus of most of the included subjects were to be physically fit and to have fun, our study population were defined as recreational athletes, consistent with the definition previously used by Laquale [10]. A detailed description of exclusion and inclusion criteria has been published previously [11]. The study subjects were asked about their habitual intake of omega-3 fatty acid supplements, including CLO supplements. C-reactive protein levels were assessed before and 24 h following the race. The study was approved by the Regional Ethics Committee (REK 2013/550) and complies with the Declaration of Helsinki. All participants signed informed consent forms prior to enrollment.

### Supplement use

Data concerning supplement use was obtained by electronic questionnaire. Supplement use was reported as either 1) no use, 2) sporadic use, 3) used only the last couple of weeks, 4) used only in conjunction with the race and 5) uses regularly. For data analysis, categories 2–4 were combined, and analyzed as sporadic use, as only a few participants reported use in category 3 and 4. No data regarding manufacturer or dosage of supplements were collected. “Regular users” was the main comparator. Contents of examples of different supplements are outlined in Table 1.

**Table 1:** The contents and differences between different omega-3 fatty acid supplements and CLO from the market leaders in Norway. The table reports the daily intake recommended by the manufacturer

	Nycoplus concentrated Omega-3^**1**^	Biopharma Norwegian Omega-3^**2**^	Biopharma Triple Omega-3^**2**^	Møller’s the original Omega-3^**3**^	Møller’s Tran (CLO)^**4**^
PUFAs	1.25 g	0.202 g	0.650 g	0.70 g	1.20 g
- DHA	0.43 g	0.096 g	0.22 g	na	0.60 g
- EPA	0.60 g	0.080 g	0.33 g	na	0.40 g
Vitamins					
- A	na	na	250 μg	250 μg	250 μg
- D	na	na	20 μg	15 μg	10 μg
- E	na	na	10 mg	10 mg	10 mg

*Na* not available. *PUFA* polyunsaturated fatty acids

^*1*^ *Contents of 2 capsules, recommended 1–4 capsules/day*

^*2*^ *Contents of 2 capsules, recommended 2–4 capsules/day*

^*3*^ *Contents of 2 capsules*

^*4*^ *Contents of 5 ml CLO. Møllers had 97% of 2017 marked share on CLO supplements, according to the manufacturer*

### Data collection

Data was collected on site the day before the race, and at 3- and 24 h following the race. Only minimal increase in CRP was observed at 3 h post-race (Supplementary Figure 1), and therefore data on CRP increase and its relation to supplement use was analyzed for the CRP increase between baseline and 24 h post-race. Data collection included blood pressure, electrocardiogram, waist circumference, body weight and blood samples. Also, online surveys were used to assess demographics, cardiovascular risk factors, training loads and training experience. Use of different kinds of supplements were also included in the online survey.

### Blood samples

Venous blood samples were drawn from the antecubital vein. Serum samples were centrifuged within 60 min at 2000 G for 10 min and analyzed within 24 h at Stavanger University Hospital. CRP was analyzed on Architect c16000TM (Abbott Diagnostics, Abbot park, Illinois, USA), using quantitative immunoturbometric determination of CRP (Multigen CRP Vario assay). Creatin kinase (CK), creatinine, low-density lipoprotein (LDL) and high-density lipoprotein (HDL) were also analyzed on Architect c1600TM (Abbott Diagnostics, Abbot park, Illinois, USA). Plasma b-type natriuretic peptide (BNP) was analyzed on Architect SR2000i (Abbott Diagnostics). Glomerular filtration was estimated (eGFR) using the CKD-EPI equation. Haemoglobin was measured using Sysmex XE-5000 (Sysmex, Kobe, Japan). These variables were included in the baseline assessment of the included subjects.

### Statistical analysis

Normally distributed continuous variables are reported as mean ± SD. Continuous variables with a markedly skewed distribution are reported as median (25th–75th percentile). The Shapiro-Wilk test was used to test for normality. A two-tailed *p*-value of < 0.05 was considered significant. Between group differences was assessed by Mann-Whitney U test or Kruskall-Wallis test, as appropriate. Multiple linear regression analysis was performed using an enter model that compared regular users of omega-3 fatty acid supplements to those who reported non- or sporadic use. Data in the multiple regression models are presented as B (95% confidence interval). Ln-transformation of the dependent variable (CRP) was performed due to skewed residuals. Adjustments were conducted based on bivariate correlations (Supplementary Table 1). Variables with marked collinearity were excluded. The following variables were assessed in bivariate correlation analysis with the CRP response: Age, body mass index (BMI), body weight, waist circumference, systolic blood pressure (SBP), diastolic blood pressure (DBP), eGFR, Framingham risk score, resting heart rate, MET hours per week, number of competitions/5 years, race duration and delta CK (baseline-24 h). Body mass index was chosen to represent body composition, and due to collinearity body weight and waist circumference were excluded. Similarly, number of competition/5 years was omitted due to collinearity with race duration. Age and sex were included into the multiple regression models due to the known impact of these variables on the body’s physiological responses. Delta CK was included due to the known association between muscle-derived interleukin 6 and CRP, and higher education was included to adjust for socioeconomic status. Sensitivity analysis are included in the supplementary appendix with multiple linear regression analysis that compares regular users of CLO to subjects who reported no use of either supplements (*n* = 612). Statistical analysis were performed using the statistical software program SPSS version 24.

## Results

Study subjects were 46.8 (40.1–52.6) years old and 783 (79.1%) were males. They finished the 91 km mountain bike race in 3.8 (3.4–4.3) hours. CRP increased from 0.7 (0.4–1.3) mg/L to 6.9 (4.0–11.8) mg/L, *p* < 0.001, 24 h following the race.

**Fig. 1:**
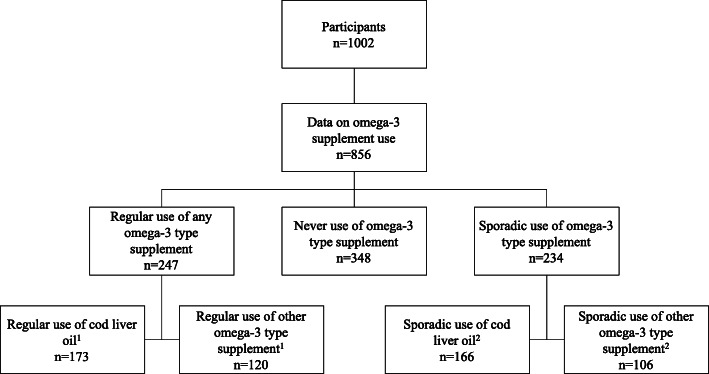
Outline of study participants and use of omega-3 fatty acid supplements. ^1^19 subjects used both cod liver oil and other omega-3 fatty acid supplement. ^2^38 subjects reported sporadic use of both cod liver oil and other omega-3 fatty acid supplement

In total, 856 (85%) of the included subjects reported data on the use of supplements containing omega-3 fatty acids (Fig. 1). Supplements were used either regularly (*n* = 274), occasionally (*n* = 234) or never (*n* = 348). Baseline characteristics of the subjects are outlined in Table 2. Regular users of omega-3 fatty acid supplements were older, had lower BMI and body weight, and reported higher training loads prior to the race than sporadic and non-users.

**Table 2:** Baseline characteristics of subjects who reported on use of omega-3 fatty acid supplements, and baseline characteristics of the sub cohorts who reported regular or sporadic use of omega-3 fatty acid supplements and the subjects who reported no use these supplements. *P*-value reflects between-group differences between regular users, sporadic users and no users. Data are reported as mean (SD) or median (25th, 75th percentile)

	All participants (***n*** = 856)	Regular use (n = 274)	Sporadic use (n = 234)	No use (n = 348)	p-value
Age, years	46.8 (40.3–58.0)	49.2 (41.8–54.7)	45.8 (39.1–51.7)	45.7 (40.0–51.1)	< 0.001
Males, n (%)	656 (76.6)	194 (70.8)	182 (77.8)	280 (80.5)	0.004
BMI, kg/m^2^	25.2 (23.7–27.4)	25.0 (23.2–26.8)	25.6 (23.8–27.9)	25.3 (24.0–27.6)	0.006
Body weight, kg	81.4 (74.2–89.1)	79.5 (71.8–87.4)	82.4 (74.3–90.5)	82.6 (75.4–89.3)	0.007
Waist circumference, cm	85.0 (80.0–92.0)	84.0 (79.3–90.9)	86.0 (79.0–93.0)	86.0 (81.0–93.5)	0.015
Systolic blood pressure, mmHg	136 (126–148)	136 (127–145)	136 (126–149)	137 (126–148)	0.67
Diastolic blood pressure, mmHg	79 (73–86)	79 (73–86)	80 (73–86)	80 (74–87)	0.58
Resting HR, beats/min	59 (53–67)	60 (54–67)	58 (52–67)	58 (53–65)	0.24
Current smokers, n (%)	11 (1.2)	3 (0.7)	1 (0.0)	7 (2.0)	0.28
Framingham risk score, %^1^	1.0 (0–5)	1 (0–5)	1 (0–4)	1 (0–4)	0.70
MET hours per week ^2^	51 (32–78)	54 (33–83)	47 (28–75)	51 (33–75)	0.009
Number of races past 5 y, n	7 (3–15)	7 (4–16)	5 (2–12)	7 (3–15)	0.072
Higher education, n (%)	532 (62.1)	166 (60.6)	158 (67.5)	208 (59.8)	0.16
**Race performance**					
Race duration, h	3.7 (3.3–4.1)	3.8 (3.3–4.4)	3.7 (3.4–4.1)	3.6 (3.2–4.1)	0.23
Maximal HR during race, bpm ^3^	178 (170–186)	176 (169–185)	179 (171–185)	178 (172–187)	0.27
Maximal HR of estimated maximal HR, % ^3^	100.4 (96.7–104.5)	100.1 (95.9–104.8)	100.1 (96.3–103.6)	100.4 (97.4–105.1)	0.67
Mean HR during race, bpm ^3^	157 (148–165)	154.0 (145.0–163.0)	158.0 (150.0–165.0)	158.0 (149.0–165.0)	0.051
Mean HR of estimated maximal HR, % ^3^	88.5 (84.5–92.5)	88.3 (83.0–92.3)	88.2 (84.4–92.5)	89.0 (85.4–92.7)	0.33
**Blood samples at baseline**					
BNP, pg/mL	13.4 (10.0–21.1)	14.3 (10.0–22.6)	14.0 (10.0–21.4)	12.5 (10.0–19.2)	0.06
CRP, mg/L	0.7 (0.4–1.3)	0.6 (0.3–1.1)	0.8 (0.5–1.3)	0.8 (0.4–1.5)	0.001
Creatinine, umol/L	83.7 ± 11.8	82.4 ± 12.5	84.3 ± 11.9	84.3 ± 11.1	0.051
eGFR, mL/min/1.73m^2^	91.2 ± 12.8	90.1 ± 13.0	91.4 ± 12.9	91.8 ± 12.5	0.33
LDL, mmol/L	3.2 (2.6–3.7)	3.1 (2.4–3.8)	3.2 (2.6–3.7)	3.2 (2.7–3.8)	0.20
HDL, mmol/L	1.5 (1.3–1.7)	1.5 (1.3–1.8)	1.5 (1.2–1.7)	1.4 (1.2–1.6)	< 0.001
Haemoglobin, g/dL	14.5 ± 1.0	14.4 ± 1.0	14.4 ± 1.0	14.5 ± 1.0	0.04

^1^ Framingham risk score: 10-year risk of death or myocardial infarction

^2^ MET = Metabolic equivalents (3.5 ml O^2^/kg/min). Estimated by IPAQ-SF

^3^ Self-reported data, available for 540 subjects (54.5%)

*BMI* body mass index, *HR* heart rate, *BNP* B-type natriuretic peptide, *CRP* C-reactive protein, *eGFR* estimated glomerular filtration rate, *LDL* low-density lipoproteins, *HDL* high-density lipoprotein

### Omega-3 fatty acid supplement use, CRP and creatine kinase concentrations

**Fig. 2:**
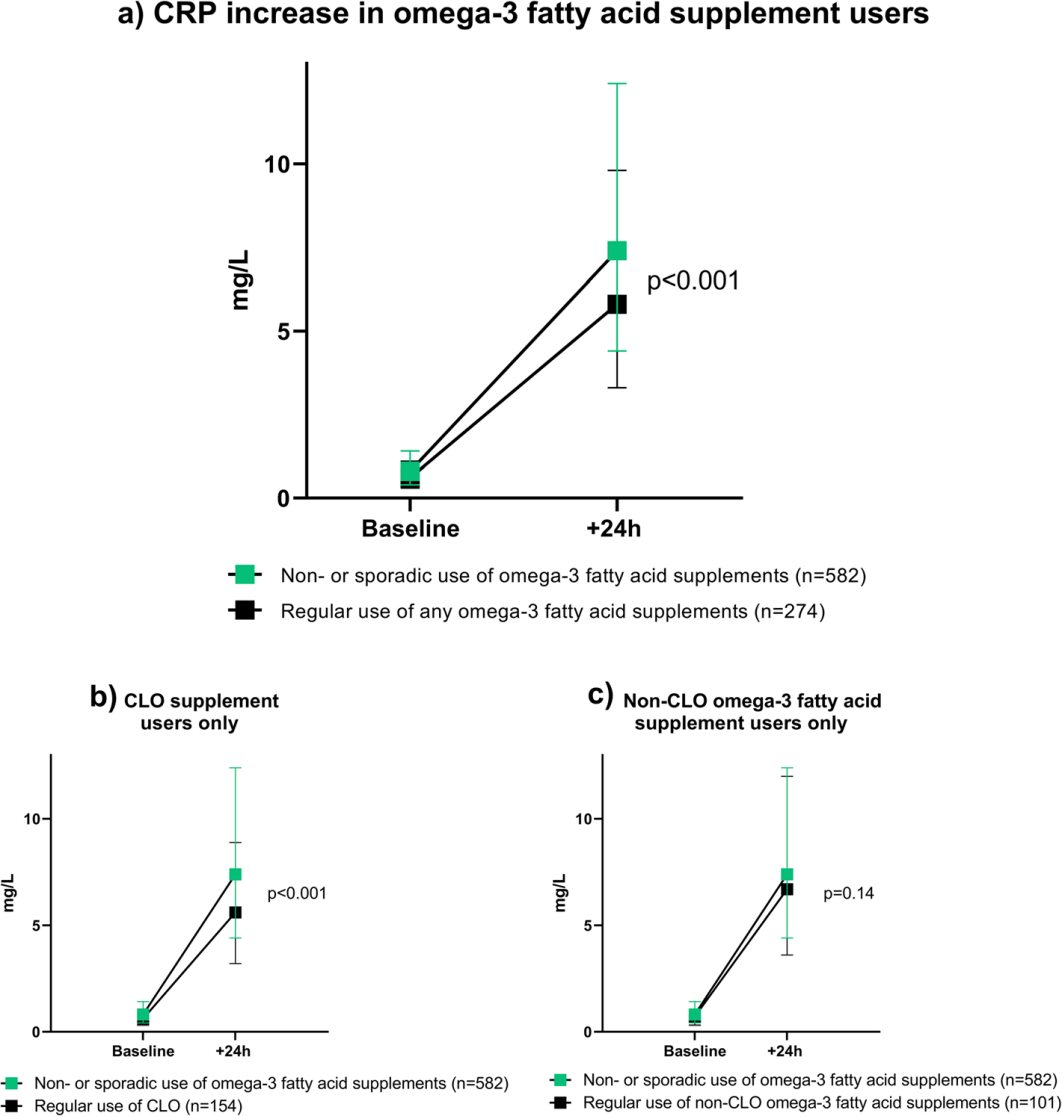
CRP increase from baseline to 24 h after the race, comparing non- or sporadic use of omega-3 fatty acids to a) regular use of any omega-3 fatty acid supplement, b) regular use of CLO and c) regular use of a non-CLO omega-3 fatty acid supplement

Subjects who reported regular use of omega-3 fatty acid supplements were found to have lower CRP-levels both before and following the race as compared to non-users (*p* < 0.001) and to non-users and sporadic users (*p* < 0.001, Fig. 2). In multiple regression analysis, regular use of any omega-3 fatty acid supplements was associated with a 16% (95% confidence interval: − 28- -5%), *p* = 0.006 decrease in ln-transformed delta CRP level as compared to subjects who reported no or sporadic use of omega-3 fatty acid supplements (Table 3, Model 4).

**Table 3:** Association between regular use of any omega-3 fatty acid supplements (n = 274) and change in concentrations of Ln-transformed CRP (B (95% confidence interval)) compared with non- or sporadic users (*n* = 582)

	Model 1	Model 2	Model 3	Model 4
CRP baseline				
Regular use of any omega-3	−25 (−27- -12), p < 0.001	−25 (−28- -13), *p* < 0.001	−18 (−30- -7), *p* = 0.002	−19 (−31- -7), p = 0.002
CRP 24 h post-race				
Regular use of any omega-3	−25 (−37- -14), *p* < 0.001	−23 (−34- -11), *p* < 0.001	−16 (−26- -5), *p* = 0.004	−16 (−26- -5), p = 0.003
Delta CRP 0-24 h				
Regular use of any omega-3	−27 (−40- -14), p < 0.001	−24 (− 27- -11), p < 0.001	−17 (− 28- -5), p = 0.006	−16 (− 28- -5), *p* = 0.006

Model 1: Unadjusted, Model 2: adjusted for age and sex, Model 3: As for Model 2, but also adjusted for body mass index and race duration, Model 4: As for Model 3, but also adjusted for delta creatine kinase (baseline-24 h post-race), MET hours per week, resting heart rate and higher education

There was a highly significant increase in creatine kinase values following the race (*p* < 0.001), but no difference between the different categories of supplement users (Supplementary Figure 2). There was a significant bivariate correlation (*r* = 0.27, *p* < 0.001) between the delta value of creatine kinase and CRP (baseline versus 24 h). In the multiple regression models, delta CK remained an independent predictor of the CRP response, but did not affect the overall results of lower CRP among regular users of omega-3 fatty acid supplements.

### Difference between omega-3 fatty acid supplements

Cod liver oil (CLO) was the most used omega-3 fatty acid supplement (*n* = 173). Users of CLO were more often male but had otherwise similar baseline characteristics as regular users of other omega-3 fatty acid supplements (Supplementary Table 2). When the CRP levels were examined in subgroups of regular users of other omega-3 fatty acid supplements (*n* = 120) or regular users of CLO, only CLO users were found to have significantly lower CRP levels as compared to non-users and sporadic users (Fig. 2). This translated into a decrease in Ln-transformed CRP levels of 24% (CI: − 38- -11) %, *p* < 0.001 following the race for regular users of CLO in the fully adjusted multiple regression models (Supplementary Table 3). Sensitivity analysis confirmed that regular users of CLO supplements had lower CRP response also in comparison to subjects who reported no use (*n* = 348, subjects with sporadic use excluded, Supplementary Table 4). Furthermore, a direct comparison within subjects who reported regular use of either omega-3 supplements (*n* = 101) or CLO (*n* = 154) were undertaken (regular users of both supplements excluded). This analysis showed a borderline significant trend in lower CRP levels for the regular users of CLO (Supplementary Table 5).

Regular use of both CLO and another omega-3 fatty acid supplement was reported in 19 subjects (1.9%), representing the group with the potentially highest intake of omega-3 polyunsaturated fatty acids (PUFAs). CRP at baseline was 0.6 (0.4–1.1) mg/L increasing to 5.8 (3.2–11.0) mg/L at 24-h following the race. No additional effect on the inflammatory response to exercise was observed in this group as compared to those who only reported regular use of CLO.

**Fig. 3:**
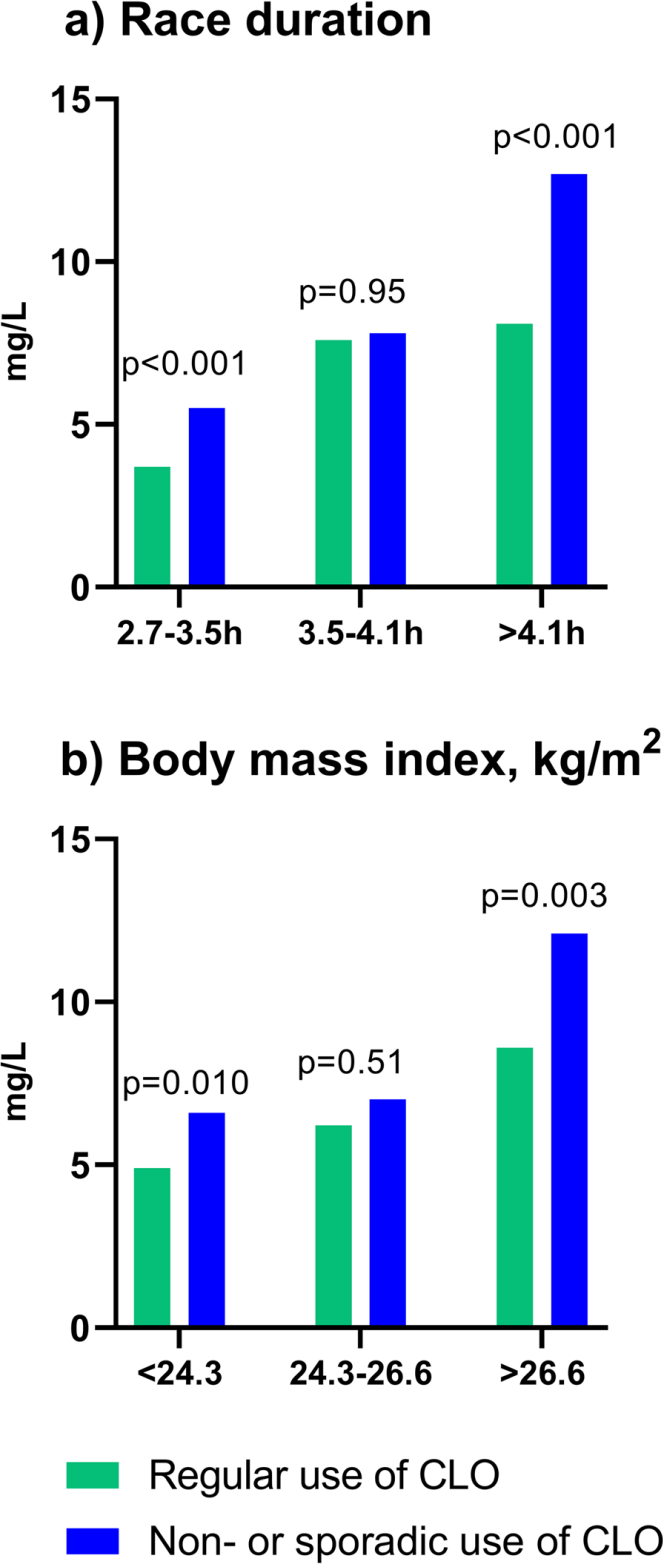
Mean delta CRP increase from baseline to 24 h after the race in subjects who use CLO regularly (green) versus the rest of the cohort (blue), divided into tertiles based on a) race duration and b) body mass index

Race duration and body mass index were also independent predictors of the CRP-response in the multiple regression models. The largest differences in delta CRP levels between regular users and non-regular users of CLO were observed for those with the highest BMI (6.2 (3.4–10.8) vs 8.5 (5.6–15.3) mg/L, *p* = 0.003) and the longest race duration (5.4 (2.9–10.9) vs 9.2 (5.1–16.5) mg/L, *p* < 0.001). However, the attenuation of the CRP response for regular users of CLO were evident across tertiles (Fig. 3).

## Discussion

This is the first large-scale study to explore and compare the effects of omega-3 fatty acid supplement use on CRP levels following the induction of a sterile inflammatory response by strenuous physical exercise in healthy individuals. There are several novel and important findings. First, use of omega-3 fatty acid supplements was common in the present cohort, with about two-thirds of subjects reporting either regular or sporadic use of these supplements. Second, subjects who reported regular use of omega-3 fatty acid supplements had lower baseline and exercise-induced CRP levels compared with other subjects. Third, in subgroup analysis only regular use of CLO was associated with lower baseline- and exercise-induced CRP levels. Fourth, in multiple regression models, the beneficial effects of regular CLO consumption, was evident in all models explored. These findings imply that omega-3 fatty acid supplements, and particularly CLO, are associated with a reduction in both basal and exercise-induced inflammation in recreational athletes.

### Use of supplements

The use of dietary supplements in the general population is common. Bailey et al. found that 49% of the general population in the US uses dietary supplements [12]. Among athletes, the use of dietary supplements seems to be even higher, but exact estimates are difficult and approximately 14% of athletes use omega-3 fatty acid supplements [13]. In Norway, there is a long tradition of CLO supplement use. The main manufacturer reports yearly sales of about 2.5 million bottles of CLO in Norway (population: 5.4 million) [14]. Three studies reporting on the use of CLO in the 1990–2000 found between 18 and 45% of their cohorts reported regular use of CLO supplements [15–17]. To our knowledge, there are no previous studies on the use of CLO in athletic cohorts.

### The CRP response in relation to regular use of omega-3 fatty acid supplement

In this present analysis, regular use of omega-3 fatty acid supplements, and in particular regular use of CLO, was associated with lower CRP levels both at baseline and following exercise. The difference in CRP levels following exercise was most pronounced in the least fit subjects and in the subjects with the highest BMI levels (Fig. 3).

The association between intake of omega-3 fatty acid supplements and inflammation has been studied in several cohorts, but the results have been conflicting. Omega-3 fatty acid supplementation has been found to reduce inflammatory biomarkers in populations with various chronic inflammatory or non-inflammatory diseases [10, 18, 19]. Importantly, the Vitamin D and Omega-3 trial (VITAL) found no decrease in inflammatory markers in subjects that used either 1 g omega-3 or 50 μg vitamin D daily for 1 year [20].

The anti-inflammatory properties of CLO are less explored. Our study showed that the anti-inflammatory effect was largest in CLO users. One earlier randomized controlled trial found that supplementation with CLO was associated with a decrease in CRP in a cohort of women with gestational diabetes. The authors attributed this to the content of omega-3 PUFAs in CLO [21]. The possible impact of the other components of CLO, such as vitamins A, E and D, were not discussed [21]. These vitamins, present both in CLO and some other omega-3 fatty acid supplements, may contribute to anti-inflammatory effects. Vitamin E is an antioxidant, which theoretically could be beneficial in neutralizing the effects of oxygen free radicals, but prior studies have been conflicting [22, 23]. PUFAs are important components of cell membranes [24]. Oxygen free radicals react more quickly with vitamin E than with PUFAs, hence the presence of vitamin E may protect the integrity of the cell membranes, in turn preventing cell damage [25, 26]. Furthermore, the vitamin A precursor, beta-carotene, and vitamin E have been associated with reduction in muscle damage and enhanced recovery from exercise [27]. Vitamin D is mostly considered to be important in regulating calcium and phosphate metabolism, however, some studies also report on anti-inflammatory effects of vitamin D supplements [28, 29]. Whether the presence of these vitamins and possible differences in vitamin content between supplements may contribute to the attenuation of the exercise-induced CRP response found in the present analysis, should be explored in future studies.

### The impact of quality, quantity and content

The observed anti-inflammatory differences between users of CLO and other omega-3 fatty acid supplements in the present study may relate to the quality of the supplement, in particular the degree of oxidation of the fatty acids [30, 31]. The Global Organization for EPA and DHA Omega-3 s has recommended a maximal limit of total oxidation value (TOTOX) of 26 [32]. Recent studies have suggested that high-quality fish oil supplements may have more favourable effects than similar doses of more oxidized PUFAs [33, 34]. More than 39% of omega-3 fatty acid supplements exceeds the limit of excess oxidation [34–36]. Moreover, in a Norwegian study of over-the-counter omega-3 fatty acid supplements the median TOTOX value in 54 available supplements was 29.0 (minimum-maximum: 8.1–113.6) [37]. The main CLO manufacturer in Norway (Möller’s, Oslo) reports a TOTOX value of 4.1 [38]. The degree of oxidation might therefore be of importance when interpreting these results.

The quantity of PUFA intake might further explain some of the difference in effect observed in the present study. As outlined in Table 1, the recommended daily dose of CLO (5 ml) contains 1.2 g of PUFAs. For other omega-3 fatty acid supplements, intake is commonly recommended as 1–4 capsules per day, making the exact dose consumed difficult to assess. Furthermore, the specific content of EPA and DHA is also not always available. Conversely, some of the omega-3 fatty acid products might yield lower PUFA intake as compared to that of CLO supplements, particularly since CLO is often consumed in a tablespoon, which contains 2–3 times the recommended daily dose [16].

EPA and DHA are usually both present in omega-3 fatty acid supplements. Emerging evidence suggests that the two fatty acids have different health benefits. In the Reduction of Cardiovascular Events with Icasopent Ethyl-Intervention Trial (REDUCE-IT), use of 4 g of PUFAs (icosapent ethyl) was found to lower the risk of ischemic events in subjects with hypertriglyceridemia. This product consists of 100% EPA, and corresponds to 10 times more EPA than the contents in a recommended daily dose of CLO [39]. DHA has also been shown to lower triglyceride levels, but might also increase levels of low-density lipoprotein (LDL), and high-density lipoprotein (HDL) [40, 41]. Furthermore, in the Comparing EPA to DHA study (ComparED), DHA and EPA were compared head-to-head and against placebo in healthy subjects with abdominal obesity and subclinical systemic inflammation, and results indicate that DHA at a dose of 3 g/day for 10 weeks may be more effective than a similar dose of EPA in attenuating inflammation [40]. However, a meta-analysis by Li et al. concluded that a higher daily dose of EPA lead to greater lowering effect on CRP in healthy subjects, as compared to DHA or omega-3 fatty acid supplements. DHA, on the other hand, was found to have a dose-dependent decrease of the level of the pro-inflammatory cytokine IL-6 [42]. These underlying differences among supplements used by the current study participants may explain some of the observed differences in effect.

In the present analysis, the least fit subjects with the highest BMI had the highest exercise-induced CRP levels. This is in line with our previous study demonstrating a strong correlation between fitness and post-exercise CRP levels [2]. In the present study, regular use of CLO was associated with lower CRP levels both at baseline and following exercise compared with all other groups. Importantly, the largest benefits of CLO use on CRP levels, were found in the least fit subjects and in subjects with the highest BMI levels (Fig. 3). Obesity is characterized by a low-grade chronic inflammation [43]. In line with our findings at rest, prior studies have demonstrated significant reductions in baseline inflammatory markers such as IL-6 (up-stream of CRP) in obese subjects receiving EPA and DHA [44, 45]. To our knowledge the present study is the first to suggest that there may be an increased beneficial effect of CLO on exercise-induced inflammation in moderately obese individuals with reduced fitness. The reason for the difference in CRP levels between CLO and other omega-3 supplements is not clear. The difference may relate to amount, quality or balance of the different PUFAs and other contents of CLO compared with the other supplements as discussed above. The present findings suggest that regular use of CLO may have a beneficial effect by attenuating post-exercise inflammation, potentially reducing the post-exercise discomfort, and thereby increasing the motivation to increase exercise in overweight and obese individuals with reduced fitness.

### Limitations

There are several limitations to the present study. The main limitations relate to the observational study design, and the lack of information on supplement manufacturer, content, duration and quantity of supplement used. In previous studies, intake of CLO has been reported to be significantly lower in people with lower socioeconomic rank and education [16]. In our material, however, 63.6% of non- and sporadic users of omega-3 fatty acid subjects reported higher education, vs 60.6% among regular users of omega-3 fatty acid supplements, *p* = 0.24.

## Conclusion

In this large-scale study on healthy recreational athletes, the exercise-induced CRP was attenuated in subjects who used omega-3 fatty acid supplement regularly. This effect was driven by the users of CLO supplements. These findings imply that regular use of CLO supplements may be associated with a reduction in both basal and exercise-induced inflammation in athletes. The association between lower exercise-induced inflammation, PUFA intake, and potential effects on recovery parameters needs to be further elucidated in future studies.

## Supplementary Information

**Additional file 1: Table S1.** Bivariate correlations (Spearman’s rho) between baseline and exercise-induced CRP and baseline- and race characteristics. **Table S2.** Baseline characteristics of omega-3 vs CLO regular users. Nineteen subjects reported regular use of both supplement and are excluded from between group analysis. **Table S3.** Association between regular use of CLO and change in concentrations of Ln-transformed CRP (B (95% confidence interval)) compared with non- or sporadic users (*n* = 612). **Table S4.** Sensitivity analysis on the association between regular use of CLO as compared to never use (*n* = 437) of CLO and change in concentrations of ln CRP (B (95% confidence interval)). **Table S5.** Association between regular use of CLO (*n* = 154) vs regular users of other omega-3 fatty acid supplements (*n* = 101), and change in concentrations of Ln-transformed CRP (B (95% confidence interval). **Figure S1.** C-reactive protein increased from baseline with maximal values measured at 24 h post-race (median with 25th–75th percentiles). **Figure S2.** Creatin Kinase increase from baseline to 24 h post-race (median with 25th–75th percentile).

## Data Availability

Data will be made available upon reasonable request.

## Abbreviations

BMI: Body mass index
BNP: B-type natriuretic peptide
CLO: Cod liver oil
CRP: C-reactive Protein
DHA: Docosahexaenoic acid
DPA: Docosapentaenoic acid
eGFR: Estimated glomerular filtration rate
EPA: Eicosapentaenoic acid
HDL: High-density lipoprotein
HR: Heart rate
LDL: Low-density lipoprotein
MET: Metabolic equivalents (3.5 ml O^2^/kg/min)
NEEDED: The North Sea Race Endurance Exercise Study
PUFA: Polyunsaturated fatty acid
REK: Regional Ethics Committee
TOTOX: Total oxidation value

## References

[1] Kasapis C, Thompson PD (2005). The effects of physical activity on serum C-reactive protein and inflammatory markers: a systematic review. J Am Coll Cardiol.

[2] Kleiven O, Bjorkavoll-Bergseth M, Melberg T, Skadberg O, Bergseth R, Selvag J (2018). High physical fitness is associated with reduction in basal- and exercise-induced inflammation. Scand J Med Sci Sports.

[3] Fatouros IG, Jamurtas AZ (2016). Insights into the molecular etiology of exercise-induced inflammation: opportunities for optimizing performance. J Inflamm Res.

[4] Lewis NA, Daniels D, Calder PC, Castell LM, Pedlar CR (2020). Are there benefits from the use of fish oil supplements in athletes? A Systematic Review. Adv Nutr.

[5] Gray P, Chappell A, Jenkinson AM, Thies F, Gray SR (2014). Fish oil supplementation reduces markers of oxidative stress but not muscle soreness after eccentric exercise. Int J Sport Nutr Exerc Metab.

[6] Tsuchiya Y, Yanagimoto K, Nakazato K, Hayamizu K, Ochi E (2016). Eicosapentaenoic and docosahexaenoic acids-rich fish oil supplementation attenuates strength loss and limited joint range of motion after eccentric contractions: a randomized, double-blind, placebo-controlled, parallel-group trial. Eur J Appl Physiol.

[7] Thielecke F, Blannin A. Omega-3 Fatty Acids for Sport Performance-Are They Equally Beneficial for Athletes and Amateurs? A Narrative Review. Nutrients. 2020;12(12). 10.3390/nu12123712 PMC7760705 33266318

[8] Calder PC (2017). Omega-3 fatty acids and inflammatory processes: from molecules to man. Biochem Soc Trans.

[9] Ridker PM (2016). From C-reactive protein to Interleukin-6 to Interleukin-1: moving upstream to identify novel targets for Atheroprotection. Circ Res.

[10] Kristensen S, Schmidt EB, Schlemmer A, Rasmussen C, Johansen MB, Christensen JH (2018). Beneficial effect of n-3 polyunsaturated fatty acids on inflammation and analgesic use in psoriatic arthritis: a randomized, double blind, placebo-controlled trial. Scand J Rheumatol.

[11] Kleiven O, Omland T, Skadberg O, Melberg TH, Bjorkavoll-Bergseth MF, Auestad B (2019). Race duration and blood pressure are major predictors of exercise-induced cardiac troponin elevation. Int J Cardiol.

[12] Bailey RL, Gahche JJ, Lentino CV, Dwyer JT, Engel JS, Thomas PR, Betz JM, Sempos CT, Picciano MF (2011). Dietary supplement use in the United States, 2003-2006. J Nutr.

[13] Knapik JJ, Steelman RA, Hoedebecke SS, Austin KG, Farina EK, Lieberman HR (2016). Prevalence of dietary supplement use by athletes: systematic review and meta-analysis. Sports Med.

[14] Tranfabrikken på Løren [Available from: https://www.orkla.no/om-oss/orkla-care/orkla-health/tranfabrikken-pa-loren/. Accessed July 2020.

[15] Brustad M, Braaten T, Lund E (2004). Predictors for cod-liver oil supplement use--the Norwegian women and Cancer study. Eur J Clin Nutr.

[16] Johansson LR, Solvoll K, Bjorneboe GE, Drevon CA (1998). Intake of very-long-chain n-3 fatty acids related to social status and lifestyle. Eur J Clin Nutr.

[17] Mai XM, Langhammer A, Chen Y, Camargo CA (2013). Cod liver oil intake and incidence of asthma in Norwegian adults--the HUNT study. Thorax..

[18] Rangel-Huerta OD, Aguilera CM, Mesa MD, Gil A (2012). Omega-3 long-chain polyunsaturated fatty acids supplementation on inflammatory biomakers: a systematic review of randomised clinical trials. Br J Nutr.

[19] Curado Borges M (2017). de Miranda Moura dos Santos F, Weiss Telles R, Melo de Andrade MV, Toulson Davisson Correia MI, Lanna CCD. Omega-3 fatty acids, inflammatory status and biochemical markers of patients with systemic lupus erythematosus: a pilot study. Rev Bras Reumatol Engl Ed.

[20] Costenbader KH, MacFarlane LA, Lee IM, Buring JE, Mora S, Bubes V (2019). Effects of one year of vitamin D and marine Omega-3 fatty acid supplementation on biomarkers of systemic inflammation in older US adults. Clin Chem.

[21] Yang S, Lin R, Si L, Li Z, Jian W, Yu Q (2019). Cod-liver oil improves metabolic indices and hs-CRP levels in gestational diabetes mellitus patients: a double-blind randomized controlled trial. J Diabetes Res.

[22] Meydani M, Evans WJ, Handelman G, Biddle L, Fielding RA, Meydani SN (1993). Protective effect of vitamin E on exercise-induced oxidative damage in young and older adults. Am J Phys.

[23] Viitala P, Newhouse IJ (2004). Vitamin E supplementation, exercise and lipid peroxidation in human participants. Eur J Appl Physiol.

[24] de Carvalho C, Caramujo MJ (2018). The Various Roles of Fatty Acids. Molecules..

[25] Burton GW, Joyce A, Ingold KU (1983). Is vitamin E the only lipid-soluble, chain-breaking antioxidant in human blood plasma and erythrocyte membranes?. Arch Biochem Biophys.

[26] Packer L (1984). Vitamin E, physical exercise and tissue damage in animals. Med Biol.

[27] Kanter MM (1994). Free radicals, exercise, and antioxidant supplementation. Int J Sport Nutr.

[28] Dadrass A, Mohamadzadeh Salamat K, Hamidi K, Azizbeigi K (2019). Anti-inflammatory effects of vitamin D and resistance training in men with type 2 diabetes mellitus and vitamin D deficiency: a randomized, double-blinded, placebo-controlled clinical trial. J Diabetes Metab Disord.

[29] Goncalves de Carvalho CM, Ribeiro SM (2017). Aging, low-grade systemic inflammation and vitamin D: a mini-review. Eur J Clin Nutr.

[30] Garcia-Hernandez VM, Gallar M, Sanchez-Soriano J, Micol V, Roche E, Garcia-Garcia E (2013). Effect of omega-3 dietary supplements with different oxidation levels in the lipidic profile of women: a randomized controlled trial. Int J Food Sci Nutr.

[31] Ottestad I, Retterstol K, Myhrstad MC, Andersen LF, Vogt G, Nilsson A (2013). Intake of oxidised fish oil does not affect circulating levels of oxidised LDL or inflammatory markers in healthy subjects. Nutr Metab Cardiovasc Dis.

[32] Nutrition GOfEaDO-satCfR (2015). Oxidation in Omega-3 Oils: An Overview.

[33] Rundblad A, Holven KB, Ottestad I, Myhrstad MC, Ulven SM (2017). High-quality fish oil has a more favourable effect than oxidised fish oil on intermediate-density lipoprotein and LDL subclasses: a randomised controlled trial. Br J Nutr.

[34] Cameron-Smith D, Albert BB, Cutfield WS (2015). Fishing for answers: is oxidation of fish oil supplements a problem?. J Nutr Sci..

[35] Jackowski SA, Alvi AZ, Mirajkar A, Imani Z, Gamalevych Y, Shaikh NA, Jackowski G (2015). Oxidation levels of north American over-the-counter n-3 (omega-3) supplements and the influence of supplement formulation and delivery form on evaluating oxidative safety. J Nutr Sci.

[36] Albert BB, Derraik JG, Cameron-Smith D, Hofman PL, Tumanov S, Villas-Boas SG (2015). Fish oil supplements in New Zealand are highly oxidised and do not meet label content of n-3 PUFA. Sci Rep.

[37] Ruyter B, Grimmer S, Thorkildsen T, Todorcevic M, Lalic M, Vogt G (2010). En screening av omega-3-oljer med hensyn til variasjon i oksidasjonsgrad, innhold av oksidasjonsprodukter og effekt på markørsystemer.

[38] Möllers tran [Available from: https://www.mollerstran.dk/om-mollers/. Accessed July 2020.

[39] Bhatt DL, Steg PG, Miller M, Brinton EA, Jacobson TA, Ketchum SB, Doyle RT, Juliano RA, Jiao L, Granowitz C, Tardif JC, Ballantyne CM (2019). Cardiovascular risk reduction with Icosapent ethyl for hypertriglyceridemia. N Engl J Med.

[40] Allaire J, Couture P, Leclerc M, Charest A, Marin J, Lepine MC (2016). A randomized, crossover, head-to-head comparison of eicosapentaenoic acid and docosahexaenoic acid supplementation to reduce inflammation markers in men and women: the comparing EPA to DHA (ComparED) study. Am J Clin Nutr.

[41] Chang CH, Tseng PT, Chen NY, Lin PC, Lin PY, Chang JP (2018). Safety and tolerability of prescription omega-3 fatty acids: a systematic review and meta-analysis of randomized controlled trials. Prostaglandins Leukot Essent Fatty Acids.

[42] Li K, Huang T, Zheng J, Wu K, Li D (2014). Effect of marine-derived n-3 polyunsaturated fatty acids on C-reactive protein, interleukin 6 and tumor necrosis factor alpha: a meta-analysis. PLoS One.

[43] Johnson AR, Milner JJ, Makowski L (2012). The inflammation highway: metabolism accelerates inflammatory traffic in obesity. Immunol Rev.

[44] Kiecolt-Glaser JK, Epel ES, Belury MA, Andridge R, Lin J, Glaser R, Malarkey WB, Hwang BS, Blackburn E (2013). Omega-3 fatty acids, oxidative stress, and leukocyte telomere length: a randomized controlled trial. Brain Behav Immun.

[45] Itariu BK, Zeyda M, Hochbrugger EE, Neuhofer A, Prager G, Schindler K, Bohdjalian A, Mascher D, Vangala S, Schranz M, Krebs M, Bischof MG, Stulnig TM (2012). Long-chain n-3 PUFAs reduce adipose tissue and systemic inflammation in severely obese nondiabetic patients: a randomized controlled trial. Am J Clin Nutr.

