# Supplementary material for: Regular consumption of cod liver oil is associated with reduced basal and exercise-induced C-reactive protein levels; a prospective observational trial: A NEEDED (The North Sea Race Endurance Exercise Study) 2014 sub-study
Source: J Int Soc Sports Nutr. 2021 Jun 28;18:51. doi: 10.1186/s12970-021-00437-1 (PMC8240263; doi:10.1186/s12970-021-00437-1)

**Supplementary Table 1**

Bivariate correlations (Spearman’s rho) between baseline and exercise-induced CRP and baseline- and race characteristics.

|  | **Ln CRP baseline** | **Ln CRP 24h** | **Ln delta CRP** |
| --- | --- | --- | --- |
| Age | ns | R=-0.08, p=0.011 | R=-0.09, p=0.005 |
| BMI | R=0.34, p<0.001 | R=0.33, p<0.001 | R=0.30, p<0.001 |
| Body weight | R=023, p<0.001 | R=0.25, p<0.001 | R=0.23, p<0.001 |
| Waist circumference | R=0.33, p<0.001 | R=0.32, p<0.001 | R=0.29, p<0.001 |
| SBP | R=0.07, p=0.04 | ns | ns |
| DBP | R=0.14, p<0.001 | ns | ns |
| eGFR | ns | ns | ns |
| Framingham risk score | R=0.07, p=0.038 | ns | ns |
| Resting heart rate | R=0.14, p<0.001 | R=0.23, p<0.001 | R=0.21, p<0.001 |
| MET hours per week | R=-0.14, p<0.001 | R=-0.25, p<0.001 | R=-0.24, p<0.001 |
| Number of competitions/5 years | R=-0.14, p<0.001 | R=-0.32, p<0.001 | R=-0.33, p<0.001 |
| Race duration | R=0.26, p<0.001 | R=0.37, p<0.001 | R=0.36, p<0.001 |
| Delta CK baseline-24h | ns | R=0.25, p<0.001 | R=0.27, p<0.001 |

**Supplementary Table 2**

Baseline characteristics of omega-3 vs CLO regular users. Nineteen subjects reported regular use of both supplement and are excluded from between group analysis.

|  | **Regular use of omega-3 (n=120)** | **Regular use of CLO (n=173)** | **p-value** |
| --- | --- | --- | --- |
| Age, years | 47.5 ± 9.6 | 48.5 ± 9.4 | 0.38 |
| Males, % | 76 (63.3) | 131 (75.7) | 0.014 |
| BMI, kg/m^2^ | 25.0 (23.2-26.8) | 25.0 (23.2-26.9) | 0.69 |
| Body weight, kg | 76.8 (70.1-87.7) | 81.3 (73.0-87.5) | 0.09 |
| Waist circumference, cm | 83 (79-89) | 86 (80-91) | 0.06 |
| Systolic blood pressure, mmHg | 135 (124-146) | 136 (127-145) | 0.56 |
| Diastolic blood pressure, mmHg | 78 (73-86) | 79 (73-85) | 0.67 |
| Resting HR, beats/min | 61 (54-69) | 59 (53-66) | 0.14 |
| Current smokers, n (%) | 1 (0.8) | 2 (1.2) | 1.00 |
| Framingham risk score, %^1^ | 1 (0-5) | 2 (0-5) | 0.08 |
| MET hours per week ^2^ | 58 (36-87) | 54 (32-82) | 0.48 |
| Number of races past 5 y, n | 6 (4-16) | 8 (4-17) | 0.35 |
| Higher education, n (%) | 53 (44.2) | 101 (58.4) | 0.047 |
|  |  |  |  |
| **Race performance** |  |  |  |
| Race duration, h | 3.9 (3.6-4.4) | 3.7 (3.3-4.4) | 0.09 |
| Maximal HR during race, bpm ^3^ | 176 (169-186) | 175 (169-184) | 0.61 |
| Maximal HR of estimated maximal HR, % ^3^ | 100.1 (95.9-104.7) | 99.8 (95.5-104.8) | 0.77 |
| Mean HR during race, bpm ^3^ | 154 (145-161) | 154 (145-165) | 0.58 |
| Mean HR of estimated maximal HR, % ^3^ | 87.9 (82.7-91.4) | 88.2 (82.9-93.0) | 0.56 |
|  |  |  |  |
| **Blood samples at baseline** |  |  |  |
| BNP, pg/mL | 14.4 (10.0-24.3) | 14.1 (10.0-22.5) | 0.51 |
| CRP, mg/L | 0.7 (0.4-1.2) | 0.6 (0.3-1.0) | 0.048 |
| Creatinine, umol/L | 81.7 ± 12.0 | 82.9 ± 13.0 | 0.39 |
| eGFR, mL/min/1.73m^2^ | 89.5 ± 14.2 | 90.5 ± 12.5 | 0.81 |
| LDL, mmol/L | 3.0 (2.84-3.7) | 3.1 (2.6-3.8) | 0.20 |
| HDL, mmol/L | 1.5 (1.3-1.8) | 1.5 (1.3-1.8) | 0.90 |
| Hemoglobin, g/dL | 14.3 ± 1.0 | 14.5 ± 1.0 | 0.06 |

^1^ Framingham risk score: 10-year risk of death or myocardial infarction.

^2^ MET = Metabolic equivalents (3.5 ml O^2^/kg/min). Estimated by IPAQ-SF

^3^ Self-reported data, available for 540 subjects (54.5 %).

BMI = body mass index, HR = heart rate, BNP = B-type natriuretic peptide, CRP = C-reactive protein, eGFR = estimated glomerular filtration rate, LDL = low-density lipoproteins, HDL = high-density lipoproteins.

**Supplementary Table 3**

Association between regular use of CLO and change in concentrations of Ln-transformed CRP (B (95 % confidence interval)) compared with non- or sporadic users (n=612)

|  |  |  |  |  |
| --- | --- | --- | --- | --- |
|  | **Model 1** | **Model 2** | **Model 3** | **Model 4** |
| CRP baseline |  |  |  |  |
| Regular use of CLO | -30 (-45- -15) %, p<0.001 | -31 (-46- -16) %, p<0.001 | -24 (-28- -10) %, p=0.001 | -24 (-38- -11) %, p<0.001 |
| CRP 24h post-race |  |  |  |  |
| Regular use of CLO | -31 (-45- -17) %, p<0.001 | -29 (-43- -16) %, p<0.001 | -22 (-34- -10) %, p<0.001 | -22 (-34- -10) %, p<0.001 |
| Delta CRP 0-24h |  |  |  |  |
| Regular use of CLO | -34 (-49- -19) %, p<0.001 | -31 (-47- -16) %, p<0.001 | -24 (-38- -11) %, p=0.001 | -24 (-38- -11) %, p<0.001 |
| Model 1: Unadjusted, Model 2: Adjusted for age and sex, Model 3: As for Model 2, but also adjusted for body mass index and race duration, Model 4: As for Model 3, but also adjusted for delta creatine kinase (baseline-24h post-race), MET hours per week, resting heart rate and higher education. | | | | |

**Supplementary Table 4**

Sensitivity analysis on the association between regular use of CLO as compared to never use (n=437) of CLO and change in concentrations of ln CRP (B (95 % confidence interval)).

|  |  |  |  |  |
| --- | --- | --- | --- | --- |
|  | **Model 1** | **Model 2** | **Model 3** | **Model 4** |
| CRP baseline |  |  |  |  |
| Regular use of CLO | -33 (-48- -17), p<0.001 | -34 (-50- -18), p<0.001 | -26 (-41- -12), p<0.001 | -27 (-42- -13), p<0.001 |
| CRP 24h post-race |  |  |  |  |
| Regular use of CLO | -33 (-47- -18), p<0.001 | -31 (-45- -16), p<0.001 | -24 (-36- -11), p<0.001 | -25 (-37- -12), p<0.001 |
| Delta CRP 0-24h |  |  |  |  |
| Regular use of CLO | -34 (-50- -18), p<0.001 | -32 (-48- -16), p<0.001 | -25 (-39- -10), p=0.001 | -26 (-40- -12), p<0.001 |
| Model 1: Unadjusted, Model 2: Adjusted for age and sex, Model 3: As for Model 2, but also adjusted for body mass index and race duration, Model 4: As for Model 3, but also adjusted for delta creatine kinase (baseline-24h post-race), MET hours per week, resting heart rate and higher education. | | | | |

## **Supplementary Table 5**

Association between regular use of CLO (n=154) vs regular users of other omega-3 fatty acid supplements (n=101), and change in concentrations of Ln-transformed CRP (B (95 % confidence interval).

|  |  |  |  |
| --- | --- | --- | --- |
|  | **Model 1** | **Model 2** | **Model 3** |
| CRP baseline |  |  |  |
| Regular use of CLO vs regular use of other omega-3 supplements | -33 (-47- -17), p<0.001 | -21 (-42- -0.5), p=0.045 | -19 (-38- -0.4), p=0.055 |
| CRP 24h post-race |  |  |  |
| Regular use of CLO vs regular use of other omega-3 supplements | -19 (-40- -1.4), p=0.068 | -20 (-41- -0.6), p=0.057 | -18 (-37- -0.5), p=0.057 |
| Delta CRP 0-24h |  |  |  |
| Regular use of CLO vs regular use of other omega-3 supplements | -22 (-44- -0.6), p=0.056 | -23 (-45- -0.03), p=0.053 | -21 (-41- -0.03), p=0.053 |

Model 1: Unadjusted, Model 2: adjusted for age and sex, Model 3: As for Model 2, but also adjusted for body mass index and race duration,

## **Supplementary Figure 1**

C-reactive protein increased from baseline with maximal values measured at 24 hours post-race (median with 25^th^-75^th^ percentiles).


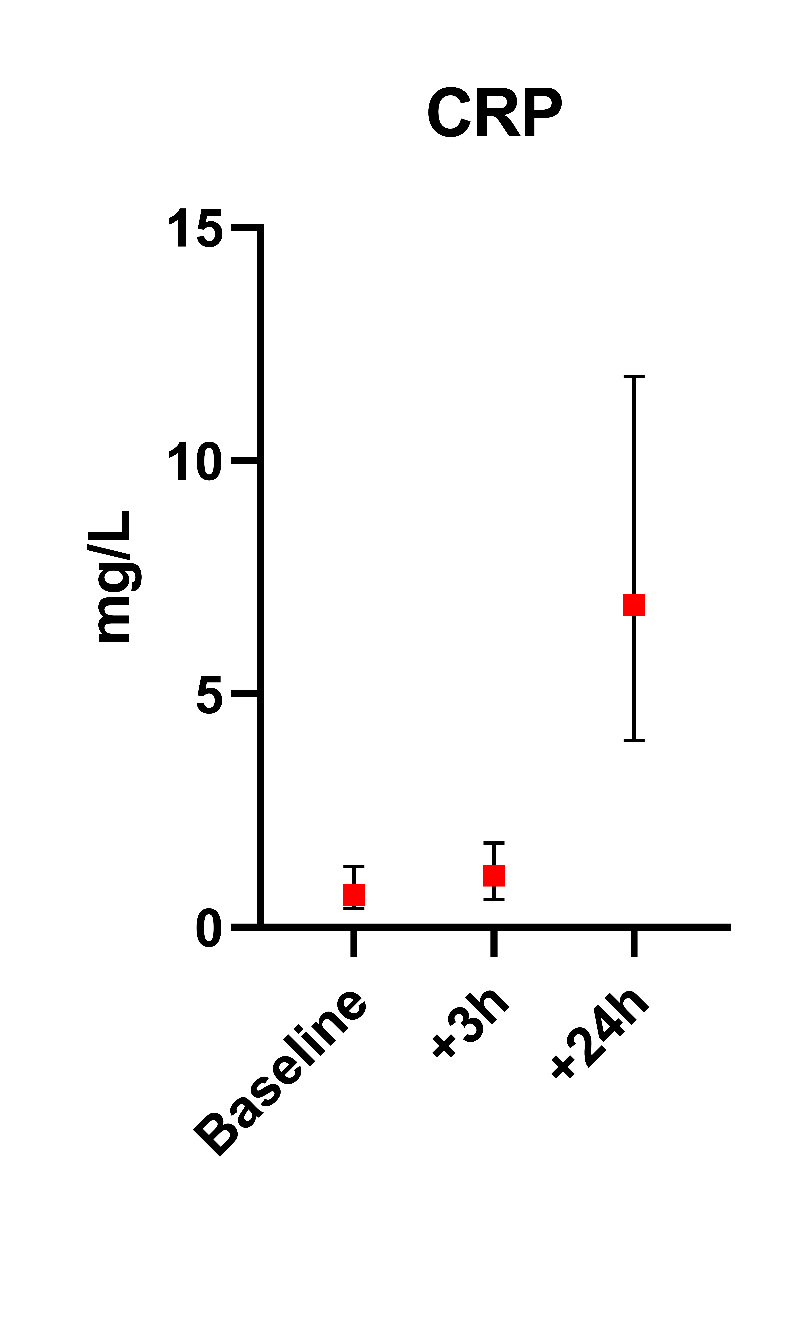


## **Supplementary Figure 2**

Creatin Kinase increase from baseline to 24 hours post-race (median with 25^th^-75^th^ percentile).


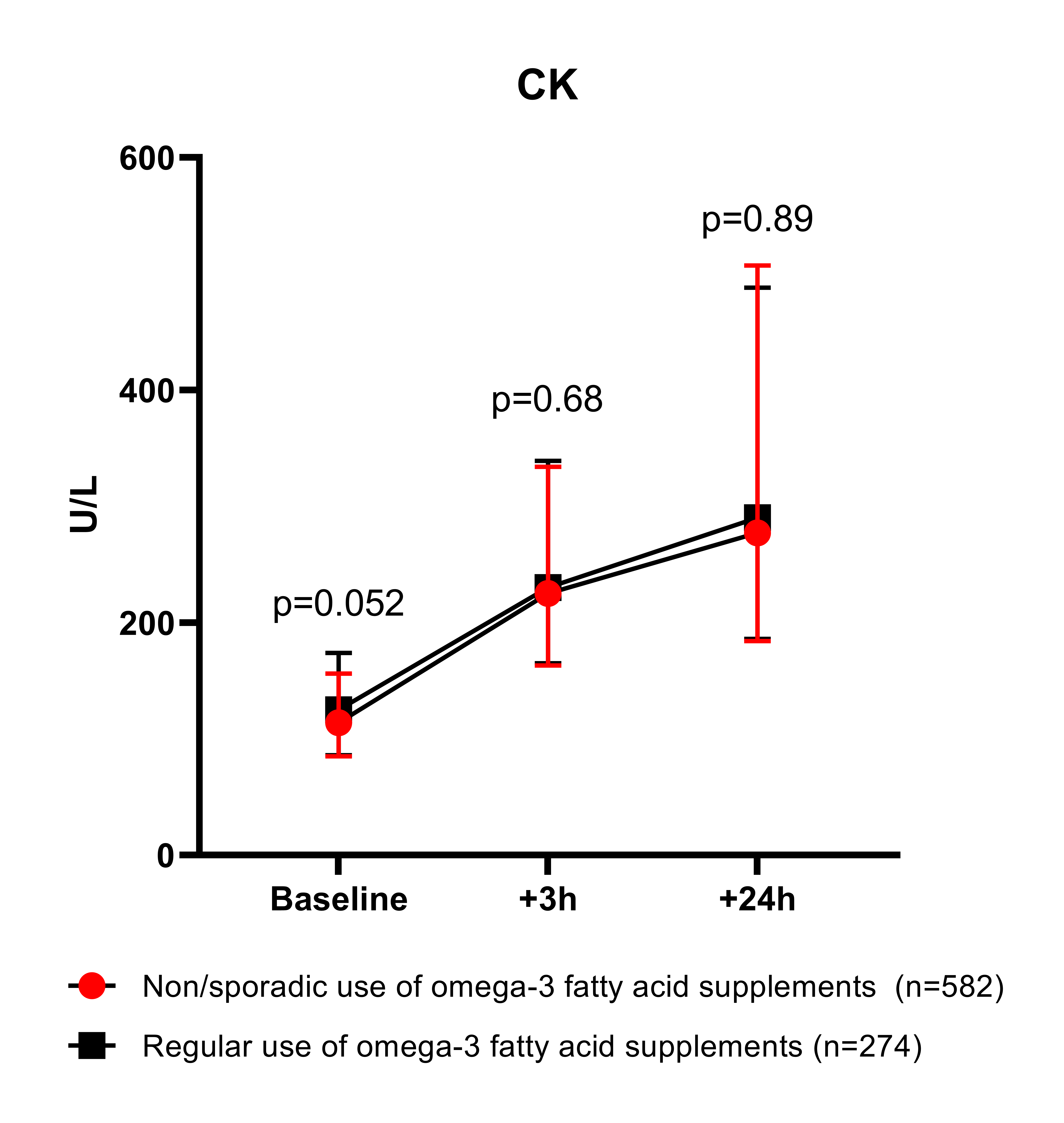

Supplement: Supplementary file 1 — Additional file 1: Table S1. Bivariate correlations (Spearman’s rho) between baseline and exercise-induced CRP and baseline- and race characteristics. Table S2. Baseline characteristics of omega-3 vs CLO regular users. Nineteen subjects reported regular use of both supplement and are excluded from between group analysis. Table S3. Association between regular use of CLO and change in concentrations of Ln-transformed CRP (B (95% confidence interval)) compared with non- or sporadic users (n = 612). Table S4. Sensitivity analysis on the association between regular use of CLO as compared to never use (n = 437) of CLO and change in concentrations of ln CRP (B (95% confidence interval)). Table S5. Association between regular use of CLO (n = 154) vs regular users of other omega-3 fatty acid supplements (n = 101), and change in concentrations of Ln-transformed CRP (B (95% confidence interval). Figure S1. C-reactive protein increased from baseline with maximal values measured at 24 h post-race (median with 25th–75th percentiles). Figure S2. Creatin Kinase increase from baseline to 24 h post-race (median with 25th–75th percentile). [file 12970_2021_437_MOESM1_ESM.docx]
